# A Descriptive Analysis of the Interactions During Clinical Supervision

**DOI:** 10.3389/fpsyg.2019.00669

**Published:** 2019-03-27

**Authors:** Mónica Novoa-Gómez, Oscar Córdoba-Salgado, Natalia Rojas, Luis Sosa, David Cifuentes, Sara Robayo

**Affiliations:** Faculty of Psychology, Fundación Universitaria Konrad Lorenz, Bogotá, Colombia

**Keywords:** clinical supervision, functional analysis, therapist training, evidence-based supervision, professional skills in clinical psychology

## Abstract

This study intends to analyze some skills trained during supervision. In it we describe sets of interactions (based on the American Psychological Association [APA], 2006; competency domains) happened between the supervisor and the supervisee during the supervision process. Interactions from twelve supervisor-supervisee dyads during asynchronous and direct, and individual and group supervision sessions were video recorded for this purpose. The recordings helped to determine, classify, and define behavioral response classes in each dyad’s interactions. Percentages of time spent in each behavior class were computed. A reliability of 95% CI [0.91, 0.95] among observers was obtained. The behavior classes in which more time was spent were assessment, intervention, and conceptualization skills. Behavior classes in which less time was spent were related to emotional and interpersonal processes. These findings are discussed by linking the evidence-based theory on supervision with the time spent in each behavior class.

## Introduction

Several studies indicate the relevance of the clinical supervisor-supervisee relationship from both, the therapeutic and the pedagogical viewpoint, within the field of psychotherapy. Clinical supervision is a setting in which therapists undergoing training develop the skills needed for psychotherapy (Falender, 2014; Martino et al., 2016; Falender and Shafranske, 2017) while client protection is guaranteed (American Psychological Association [APA], 2015), and the quality of service is optimized (Reiser and Milne, 2014).

In psychology, the clinical supervision is currently known as an independent professional research-based specialization, aiming to identify best practices along with specific skills required (Borders et al., 2014; American Psychological Association [APA], 2015). The literature and research in this area have increased with such recognition (Falender, 2014). It has additionally fostered the development of therapeutic models that promote systematic supervision to address practice needs (Falender and Shafranske, 2004; Myers and Sweeney, 2008; Plakun et al., 2009; Tsai et al., 2009; Rodolfa et al., 2013; Holt et al., 2015).

Several measurement and cognitive behavioral therapist training strategies rely on the supervisor-supervisee interaction (Milne et al., 2002). This is expected because this interaction constitutes the vehicle to train skills in supervisees (Cheon et al., 2009), and set the stage for learning to occur (Milne, 2018). Management and support of emotional processes related to psychotherapy and supervision itself are of capital importance within these interactions (Falender and Shafranske, 2004; Watkins, 2014; Milne and Reiser, 2017; Milne, 2018). This has led to the development of conceptualizations of the supervisory relation and of the supervision that includes emotional processes. Palomo et al. (2010) developed the supervisory relationship questionnaire to characterize and measure the relevant dimensions of the supervisory relationship. This instrument includes emotional aspects of the supervision, in a factor that explains most of the variance called “secure base.” According to Milne (2018), possible actions that promote this secure base are: empathizing and connecting emotionally, offering warmth and respond to learners needs, among others. Likewise, Milne et al. (2011a) developed an observational instrument to measure the supervision competence based on his evidence-based clinical supervision (EBCS) model that includes a component called “relating” (Milne, 2018). The inclusion of this component in this assessment tool shows its relevance, since this instrument was the strategy chosen by Milne to operationalize the clinical supervision. This component involves the supervisor’s interpersonal effectiveness and illustrates the importance that emotional processes have within supervision.

There is a parallelism between supervision and psychotherapy. In both cases, the relationships between the supervisor and the supervisee and between the therapist and the client play a major role in the process. Furthermore, the skills required for supervision benefit the therapist training and the client protection. Thus, the competences that a supervisor should have and the ones that should be developed by the supervisee have similarities (see American Psychological Association [APA], 2006, 2015). Emotional skills are an essential part of the relationship skills, in both cases, according to guidelines for supervisors and clinical therapists. For example, the therapists’ interpersonal expertise involves interpreting non-verbal and verbal cues, empathetic responding to implicit and explicit concerns. The supervisory relationship associated with the supervisor’s competencies include favoring an environment that promotes self-disclosures, and skills to repair ruptures of the supervisory relationship. The parallelism between therapist and supervisory competencies offers an opportunity for modeling therapist skills during supervision.

This paper aims to describe interactions between supervisors and supervisees relying on the evidence-based clinical practice guidelines (American Psychological Association [APA], 2006) that include the set of competencies that a clinical therapist should develop, including interpersonal skills. We will illustrate these domains of skills below.

Table 1 shows that therapists must have the skills required to provide clinical services (items a, b, and c). Therapists should seek training, be informed about the latest literature in the field, acknowledge their limitations, and use additional resources to guarantee an appropriate service (items d, e, and f). Therapists should also pursue proper training for developing skills to work with diverse population. In other words, they should be able to identify individual differences based on gender, sexual orientation, race, culture, religion among other variables. Therapists could then adapt therapy to the needs deriving from these factors (g). In addition, it is paramount the development of interpersonal skills (i.e., establishing an adequate relationship and being able to repair it when necessary) (h).

**Table 1:** Evidence-based clinical practice areas of expertise according to APA guidelines.

Components of clinical expertise American Psychological Association [APA], 2006)
Assessment, diagnostic judgment, systematic case formulation, and treatment planning (a)
Having a cogent rationale for clinical strategies (b)
Clinical decision making, treatment implementation, and monitoring of patient progress (c)
Appropriate evaluation and use of research evidence in both basic and applied psychological science (d)
Understanding the influence of individual and cultural differences on treatment (e)
Seeking available resources (e.g., consultation, adjunctive, or alternative services, etc.) as needed (f)
Diversity skills (g)
Interpersonal expertise (h)


Supervision has also been studied within particular *behavior analytic therapeutic model*s (Tsai et al., 2009). It is well-known the applications of behavior analysis (BA) to contexts of clinical therapy. BA employs learning principles to treat clinical problems and to discover strategies to alleviate them (Ghaderi, 2007; Leaf et al., 2016; Welch and Polatajko, 2016). BA takes the so-called “operant behavior” principles to apply them to therapy contexts. This application involves direct observation of behavior, development of case conceptualizations, and interventions to modify behavior during therapy, introducing procedures such as contingencies, reinforcement, shaping, etc.

As one of the therapies based on BA, FAP relies on the assumption that the relationship between the client and the therapist is the vehicle of behavior change and learning (Kohlenberg et al., 2002). FAP offers the possibility for the therapist to address different situations by safely selecting and adjusting treatments or techniques to each client (Kohlenberg et al., 2002). According to a behavior analytic approach, creating or establishing adequate contingencies for the client’s issues constitutes the foundations of any intervention, in FAP those contingencies might occur within the client-therapist relationship (Kohlenberg et al., 2002).

A similar rationale has been used to develop clinical skills. For example, Follette and Callaghan (1995) employed a therapist training based on FAP by using one-way mirror observation so the supervisor could indicate to the supervisee if their performance was effective, non-effective, or neutral. Supervisees improved their skills to identify their mistakes during intervention, and behaviors that are valuable but not frequently employed (for a similar and more modern approach see Carmel et al., 2016).

Nevertheless, other methods are usually employed during supervision. According to Milne et al. (2011b), live or video-recorded feedback, role-play and modeling proved to be the most common methods among supervisors. Both, feedback and modeling, are key elements within evidence-based supervision (Martino et al., 2016). Milne and Reiser (2012) present an evidence-based method that works not only for therapy contexts but also for therapist training. This method aims to improve the effectiveness of training and intervention by considering the main needs and the most influential elements according to literature (Milne et al., 2011b). The training includes experiential learning methods. Its efficacy can be increased by a combination of a functional BA during training and feedback from the trainee (Milne et al., 2002).

All these strategies depend on the interactions within the supervisory relationship. For this reason, understanding the different types of interactions between the supervisor and the supervisee is critical for comprehending and improving the effectiveness of the supervision (Tsai et al., 2014). In the literature, there is a supervision model for FAP training that relies on the supervisor-supervisee relationship (Callaghan, 2006b; Tsai et al., 2009). According to this model, supervisors use the supervisory relationship to improve therapists’ interpersonal repertoires (see Barraca Mairal, 2009).

Given the importance of the supervisory relationship in the FAP model, Callaghan (2006b) developed the functional assessment of skills for interpersonal therapists (FASIT) as a tool for implementing five response classes involved in interpersonal relationships. These response classes are: (a) assertion of needs, (b) interpersonal feedback, (c) management of conflict, (d) disclosure of personal information, and (e) emotional expression. Response classes a, b and e involve emotional processes, since they require appropriate regulation and expression of emotions. We used this implementation in this study because it involves many elements regarded as important in the literature.

Based on the supervisor-supervisee interaction, it is possible to expect the supervisee begins to take appropriate actions during the psychotherapy practice, as the behavior promoted by the supervisor when interacting will influence the supervisees’ interventions (see Callaghan, 2006a). For example, if the therapist needs to discuss difficult emotions avoided by the client (e.g., the sadness that accompanies the loss of someone important for them) but frequently postpone this discussion (because this aspect is also personally challenging for the therapist), the supervisor may talk to the therapist about the feelings that arise when talking about this topic with the client. In this case, the therapist avoids examining his feelings, similarly to the client. Therefore, the supervisor may address this issue during supervision to teach the therapist how to relate to the client’s emotions differently, providing a more effective intervention. This interaction is well suited to address the emotional aspects of supervision that we mentioned earlier in this paper.

Within FAP supervision, the training focus are the specific therapist’s classes of behavior regarding interpersonal repertoires. There is limited knowledge regarding other response classes that might occur during supervision, which are unrelated to the interpersonal relationship. Literature has addressed other interaction elements. However, interactions between supervisor and supervisee have not been studied using American Psychological Association [APA] (2006) guidelines for evidence-based therapy. Therefore, inquiring into the response classes that currently occur within clinical supervision is essential.

A previous research of this group studied the supervisors’ opinions about certain skills that should be developed by supervisees. However, as this information relies on the supervisors’ verbal reports, it might be biased because of social desirability reasons. This study aims to correct this matter by providing a comprehensive analysis of the kind of skills trained during supervision. Accordingly, we describe the response classes that take place between the supervisor and the supervisee along with the supervision process intended for supervisees to develop clinical skills. Skills suggested by American Psychological Association [APA] (2006) were organized into response classes for this purpose. Supervisor-supervisee interactions occurring during supervision were observed in order to determine the frequency of these response classes.

## Materials and Methods

### Participants

The sample of the study was composed by nine supervisors and one of their supervisees, working for 3 universities in Bogotá, Colombia. Fifteen video recordings of individual, direct, and asynchronous supervisions were conducted within the universities that offer undergraduate or graduate programs in clinical psychology. In most of the cases, the supervisions lasted for 1 h.

### Instruments

Following the domains of competency proposed by the American Psychological Association [APA] (2006), it was designed a *supervisor-supervisee interaction assessment manual* as an instrument to measure the response classes described by Callaghan (2006a). There were also carefully classified the interactions intended to develop skills in the supervisee. Each category comprises a response class selected based on their impact and relevance to the development of the therapist skills. Table 2 shows the 13 chosen categories.

**Table 2:** Brief description of coding categories.

Category	Description
Self-Evaluation skills	Supervisee’s assessment of their own abilities and limitations
Diversity skills	Identification of the client’s particularities by taking into account individual variables
Feedback	The supervisor describes one of the supervisee’s skills in order for him/her to improve it
Conflict management	Descriptions of conflict situations between the supervisor and the client, or between the supervisor and the supervisee or instruction to manage those situations.
Self-Revelation/experience, and emotional expression	Reports of feelings and impressions of the therapeutic process, or of the supervision process
Interpersonal proximity	Discussions or activities that promote developing skills to strengthen the therapeutic bond
Ethical skills	Favoring decision making and acting according to legal and ethical considerations
Expression of needs	Activities that encourage expressing the therapist or the client’s needs during the supervision process
Autonomy	Actions that foster autonomous decision making by the supervisee
Bidirectional communication	Activities that raise awareness of the influence the therapist behavior has on the supervisor or on the client
Conceptual management skills	Activities that promote relating case elements to theory and psychological concepts
Intervention skills	Activities that encourage an adequate use of techniques for changing and promoting the client’s wellbeing
Assessment skills	Activities that favor diagnosing and understanding the issues, in addition to assess therapeutic change


Two observers coded the interactions in each session using the classes definitions in the manual. The total length of the session was divided into 3-min intervals. Observers paused the recording at the end of each interval to individually assess and code the observed interaction. It was possible to code several types of interactions within the same interval since they were not mutually exclusive. Observers coded whether the supervisor or the supervisee started the interaction, as this proves to be relevant for identifying the efforts made by the supervisor to teach a skill required by the supervisee.

There were recorded four supervisions (not included in the final sample) to assess the definitions in the manual. Observers discussed their impressions and hesitations regarding these definitions after every observation interval. Whenever disagreements occurred, they were discussed with the two first authors of the article, who have clinical supervision experience, and defined the categories for observation. The definitions were subsequently adjusted and the second author provided observers with four training sessions for the implementation of the manual.

### Procedure

This research was reviewed and approved by the ethics committee of the Faculty of Psychology at Fundación Universitaria Konrad Lorenz. All participants provided written informed consent. Supervisors were asked not to mention any data during supervisions that could lead to the identification of the supervisees’ clients. An undergraduate student conducted the recording. The student remained in the supervision session monitoring the quality of the recording without interfering.

The videos, along with the consent forms, were stored in a safe place. Afterward, four observers that received the training, manually coded the video-recordings. Once the coding was completed, observation records were digitized for their analysis. Lastly, manually written records were filed.

## Results

The interval record used in this study allows us to determine the approximate percentage of time spent in each interaction per session. Reliability analyses of the measure of supervisor-supervisee interactions during clinical supervision were conducted. Interclass correlations were used to find the observers agreement when calculating the percentages of each session since this strategy let us find interobserver-agreement with a continuous variable. Each session agreement was used to perform a bootstrap analysis. The approximate index rate of agreement found was 0.94, 95% CI [0.91, 0.95].

Afterward, the average of the percentages of each category was calculated, and a bootstrap analysis was conducted in order to find the estimated average and the confidence interval. For this analysis, the interactions the supervisor started were differentiated from the ones the supervisee began, aiming at identifying potential variations.

**FIGURE 1:**
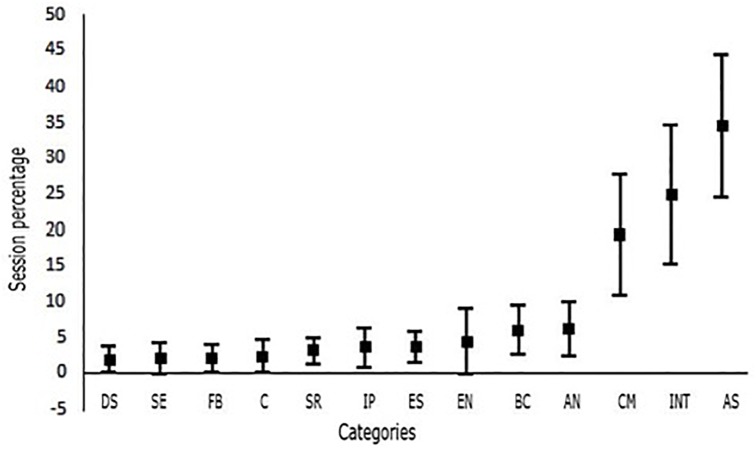
Percentages of the interactions started by the supervisor in each category using bars to represent confidence intervals at 95% level of confidence. DS, diversity skills; SE, self-evaluation; FB, feedback; C, conflict management; SR, self-revelation; IP, interpersonal proximity; ES, ethical skills; EN, expressing needs; BC, bidirectional communication; AN, autonomy; CM, conceptual management; INT, intervention skills; AS, assessment skills.

Figure 1 shows the interactions began by the supervisor during the supervisee’s training. Skills related to the task of the psychologist during therapy have a higher occurrence percentage whenever the supervisor starts the interaction. These interactions are intended to develop assessment, intervention, and conceptual management skills. In addition, these are the only skills with a higher percentage of spent time with 95% confidence. The remaining interactions are not highly distinguishable from each other and occur 10% of the time at the highest, with *autonomy* at the upper limit. Categories composing this group include interpersonal closeness, autonomy, ethical, diversity skills, and feedback. The last group comprises response classes with limited occurrences (average around 0%). These interactions aim at the supervisee’s assessment of their own behavior, skills, and limitations during the intervention or the supervision process.

**FIGURE 2:**
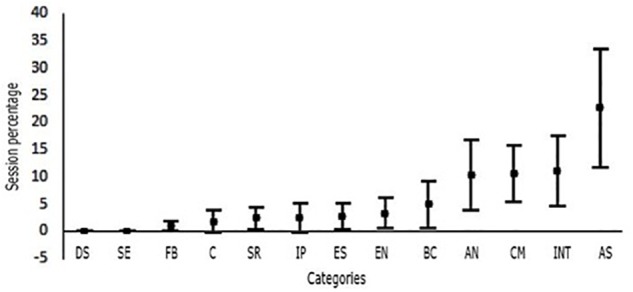
Percentages of the interactions started by the supervisee in each using bars to represent confidence intervals at 95% level of confidence. DS, diversity skills; SE, self-evaluation; FB, feedback; C, conflict management; SR, self-revelation; IP, interpersonal proximity; ES, ethical skills; EN, expressing needs; BC, bidirectional communication; AN, autonomy; CM, conceptual management; INT, intervention skills; AS, assessment skills.

Figure 2 shows the interactions started by students. As before, the group of skills related to the tasks of the psychologist within the therapy process has a higher occurrence percentage. However, only *assessment skills* class is distinguishable from other classes. Conceptual proficiency, intervention, and autonomy skills comprise between 3 and 27%, but are only different from categories occurring with a percentage around 0 which are conflict management, feedback, and emotional expression skills. The remaining interactions occur between 0 and 16% of the time. However, they are not mutually distinguishable in terms of spent time. They comprise ethical, diversity, and interpersonal skills.

## Discussion

### Clinical Psychologist Skills

Skills related to the clinical therapist duties have been extensively conceptualized. The Cube Model (Rodolfa et al., 2005) is useful to address this matter. This approach divides professional skills into two groups: foundational skills, which refer to the therapist’s professionalism underlying practice, and functional skills, which are linked to the practical aspects of the work as a therapist (Rodolfa et al., 2013). *Assessment and intervention skills* are included in the last group. This study found that interactions aiming at developing those skills had a higher occurrence percentage than the rest. Assessment skills were particularly high within interactions started both by the supervisor and the supervisee. Such findings are similar to those of Rodolfa et al. (2013). Rodolfa et al. (2013) consider that assessment and intervention-related clusters are fundamental for clinical practice. This might be because assessment skills are used throughout the entire therapy process. According to American Psychological Association [APA] (2006), these skills facilitate establishing a diagnosis, selecting the type of treatment, assessing therapeutic change, making clinical decisions to modify treatment, and seeking for other alternative resources that improve the service provided.

The percentage of self-evaluation activities during the supervision session was very low. According to Karel et al. (2014), this skill is fundamental because supervisors notice more issues in the supervisees’ performance than those perceived by themselves. This is expected because supervisees tend to overestimate their skills. Despite the encouragement to develop this skill in several discussions about supervision (Falender, 2014), this study exposes a difficulty to link scholar discussions about supervision with its actual practice, which is consistent to Falender and Shafranske’s (2017) considerations.

In addition, this study found that both, supervisors and supervisees, begin a limited number of interactions aiming at fostering the supervisee’s autonomy. Comparably, Rodolfa et al. (2013) reported that the Evidence-Based Decision-Making/Critical Reasoning cluster was frequently considered as a non-crucial skill for achieving positive results for the client. This cluster also included choosing relevant literature, its critical review and linking it to the assessment process. The minor importance given to this skill during the supervision, contrasts with the significance given to it by the American Psychological Association [APA] (2006) evidence-based practice guidelines. This inclination is alarming because if this skill is not considered significant and, thus, is not developed by the supervisee, it is possible they would not have the foundations to autonomously choose the most appropriate treatment, make clinical decisions, and modify the treatment in order to adjust it to each client. All these competences are essential for conducting autonomous practices (Milne and Reiser, 2012). For, if this skill is not systematically developed, supervisees might base their decisions on improper strategies, such as allowing biases or overgeneralizations to unconsciously influence their decisions (American Psychological Association [APA], 2006; Cummings et al., 2015).

### Fundamental Therapy Knowledge

Theoretical and conceptual knowledge required by the supervisees is paramount within supervision. This is evident because this skill shows higher percentages than the rest, specifically considering interactions started by supervisors. These findings align with the supervision model presented by the FAP (Callaghan, 2006b), the Cube Model for Competency Development (Rodolfa et al., 2013), and the Evidence Based Clinical Supervision Model (Milne and Reiser, 2017). They consider developing theoretical and conceptual skills either as a supervision goal or as a stage for linking them to the clinical skills developed throughout the supervision process (Milne et al., 2011b; Holt et al., 2015; Watkins et al., 2015). It is worth highlighting that this skill needs to be coherent with the supervisee’s approach because this is how their decisions will be guided during therapeutic intervention (Lewis et al., 2014; American Psychological Association [APA], 2015). Additionally, according to Callaghan (2006b) and American Psychological Association [APA] (2015), this skill constitutes a professional criterion within the training process because it encourages autonomous decision-making by the supervisee.

Due to the size of the study sample, it is not possible to distinguish among different stages of the supervision training. It is expected that conceptual aspects in which therapy is based require less time dedication as the supervision goes on, and the supervisees develop these skills. To continue working on this skill during the final training stages, would imply a bias obstructing the supervisees’ improvement. It is therefore significant for further research to inquire into the change of focus along the supervisees’ training.

Moreover, other related skills such as legal knowledge and ethical case management, and diversity skills occupy limited supervision time. These findings can be interpreted in two directions. First, ethical knowledge is required for clinical practice. Thus, students need to address this topic during their first training stages. Consequently, addressing it during supervision should only be necessary whenever particular dilemmas or special ethical considerations arise. Second, these skills might not be incorporated due a lack of supervision training. De las Fuentes et al. (2005) and Hatcher et al. (2013) argue that ethics –as a fundamental skill of psychological practice– should occupy a broad space within the process. Therefore, exercises and activities designed to identify and solve legal and ethical dilemmas (based on reviewing both scientific data, and current regulations) are necessary for students to take coherent positions with the profession and with the client’s wellbeing. Hence, ethical standards are as significant as scientific skills (knowledge and methodologies).

In addition to that, diversity skill training is especially significant considering that immigration from other parts of the country to Bogotá is common. For this reason, the reduced percentage of time spent during supervision to develop this skill might indicate that awareness regarding the need to address multicultural skills should increase. Such skills would improve adjustment processes of migrants arriving to the city, particularly of those coming from rural areas and moving permanently or temporarily to urban areas (Salas-García et al., 2016). Bastidas-Bilbao and Velásquez (2016) mention that supervision, as an educational program, works as an open system constantly being influenced by the socio-historic conditions of each specific location and by the group of people interacting within that place. Our findings differ from the results of Rodolfa et al. (2013) because the Interpersonal and Cultural Competence cluster was reported as one of the most significant. However, it has been found that despite of the fact that supervisors reported discussing diversity aspects, supervisees indicated that this was infrequent, and that often it was not well received when they proposed the topic during the supervision session (Jernigan et al., 2010; Falender, 2014). The supervisees’ reports in these studies coincide with the findings of this study.

### Interpersonal Relationship Skills

A general lack of dedication in developing the supervisees’ interpersonal skills was identified. For instance, conflict management, bidirectional communication, and interpersonal proximity skills occurred at an average below 5%. Conflict and feedback interactions started by the supervisees had 0 occurrences. This is remarkable because both of these skills are required to establish adherence and a good therapeutic relationship. Duff and Shahin (2010) mention that conflict is an inevitable but essential part of any working relationship. Thus, conflict management skills are fundamental. Inadequate conflict management can produce dissatisfaction with the work and the supervision, which could limit the supervision’s effectiveness and jeopardize the client’s wellbeing. Therefore, the conflict should be addressed in a way that solutions are found based on relationship aspects like trust, respect, rapport, and empathy. This will allow a productive supervision, and a constructive learning experience. Regarding interpersonal skills, Watkins and Scaturo (2013) emphasize that aspects like a safe environment, empathy, authenticity, remoralization, session planning, and appropriate affective experiences (transmitted to the supervisee through the working relationship) take part in establishing and keeping a supervisor-supervisee relationship. These elements are crucial for developing the required skills for future professional practice. Lastly, an appropriate supervisor-supervisee relationship generates an adequate therapeutic relation (Callaghan, 2006b). This has been recognized as one of the non-specific elements that contributes the most to therapeutic change (Ellis, 1991).

Regarding interactions started by the supervisor, bidirectional communication is the skill with most occurrences within this group. Interactions focused on it aim at incorporating discrimination training or efficient supervisee responses toward the impact they have on other people. This facilitates establishing a therapeutic relationship with the client or a professional relationship with the supervisor (Callaghan, 2006b). Consequently, an authoritarian relationship with the supervisor is incompatible. These findings are encouraging because, according to numerous authors, authoritarian supervision strategies are negative aspects of supervision, and obstruct the supervisee’s progress and learning (Ramos-Sánchez et al., 2002; Herschell et al., 2010; Grant et al., 2012). However, this type of interaction has a low number of occurrences, which implies that more attention should be drown to its use and development.

### Comparison With Other Supervision Interaction Measurements

Examining supervision evaluation based on cognitive behavioral therapy (CBT) and EBCS approaches provides several methods to assess supervision (Milne et al., 2013; Newman et al., 2016). Assessment criteria include, first, the client’s satisfaction with the therapeutic process and the results of the therapy (Grossl et al., 2014), second, the supervisee’s satisfaction and their skills assessment (Cliffe et al., 2014; O’Donovan and Kavanagh, 2014; Calvert et al., 2017), third, the assessment of specific supervisor skills (Bambling and King, 2014; Berger et al., 2014; Gonsalvez and Calvert, 2014; Newman et al., 2016), and lastly, the analysis of the behavior during the supervision session (Milne et al., 2002) through a systematic observation approach (Milne and Reiser, 2011).

The instrument used for this research is aligned with Milne and Reiser’s (2011) recommendations. First, it is properly designed. Second, it is optimal for application because it only requires a short training and it is clearly understood and interpreted. Finally, the findings collected with this technique are useful since they allow identifying the frequency of the supervision subjects’ interactions related to specific skills. Corrective feedback of the supervisor and the supervisee’s performance can be provided using this frequency report, according to observational data from both participants’ skills (Milne and Reiser, 2011). Hence, it can be combined with other complementary tools in order to determine if supervision lines up with the supervisee’s learning needs. The test-retest properties of this tool still need to be assessed. However, it had an adequate reliability among raters.

### Study Limitations and Future Directions

This study identified objectively the time spent developing different types of skills required for evidence-based clinical psychology, which is an advantage over other investigations based on the supervisors’ oral reports (e.g., Rodolfa et al., 2013). This identification can lead to implementing corrective actions by supervisors and university’s regulations. However, due to the transversal analysis carried out, the study did not examine the different stages of the supervisee’s skill development, for instance, half-yearly. It is, therefore, relevant that further studies examine the different stages of supervision training in order to identify potential supervision content adjustments, and interaction elements determining this content. A functional analysis is crucial to recognize precedents and consequences of relevant interaction behavior. Finally, theory and analysis in this paper were based mainly in supervision models and theory akin to behavioral therapies which might limit the generalization of this analysis to other theories and therapies.

## Author Contributions

MN-G and OC-S developed the study concept and design, critically reviewed, and corrected the manuscript. NR, LS, DC, and SR contributed with data collection and analysis and drafted the manuscript. All authors approved the final version of the manuscript and agreed to be accountable for all aspects of the work.

## Conflict of Interest Statement

The authors declare that the research was conducted in the absence of any commercial or financial relationships that could be construed as a potential conflict of interest.

## Abbreviations

APA: American Psychological Association
FAP: functional analytic psychotherapy
